# How to consider the indication of implantable cardioverter‐defibrillator in the elderly patients

**DOI:** 10.1002/clc.24201

**Published:** 2023-12-12

**Authors:** Naoya Kataoka, Teruhiko Imamura

**Affiliations:** ^1^ Second Department of Internal Medicine University of Toyama Toyama Japan

To the Editor,

Lewenhardt et al. conducted an inquiry into the clinical ramifications of implantable cardioverter‐defibrillator (ICD) implementation in elderly cohorts.^1^ They delineated the appropriateness of ICD therapy predeath as a benchmark for “benefit of ICD implantation,” juxtaposed against instances of death from any cause sans appropriate ICD therapy, termed as “no benefit of ICD implantation.” Their investigation revealed that primary prophylactic ICD implantation in elderly patients did not invariably yield advantageous outcomes. However, secondary prophylactic ICD implantation demonstrated acceptability across all age groups.^1^

Cardiovascular implantable electronic devices (CIEDs), inclusive of ICDs, bear the potential for device‐related infections. Notably, younger patients exhibit a heightened risk of enduring such infections during prolonged periods of support with CIEDs.^2^ It may be prudent to consider the detriment of such CIED‐related complications alongside the potential benefits of ICD implantation.

Significant distinctions in several baseline characteristics were evident among the three delineated groups: benefit group, no benefit group, and neutral group.^1^ Approximately 50% of the benefit group exhibited a history of myocardial infarction. Patients with an ischemic etiology tend to display improved survival subsequent to primary prophylactic ICD implantation.^3^ Meanwhile, one‐quarter of the no benefit group presented chronic obstructive pulmonary disease (COPD) and received loop diuretics. COPD stands as a major contributor to noncardiovascular mortality. The utilization of loop diuretics correlates with deteriorating renal function and heightened susceptibility to CIED‐related infections.^2^ Beyond age considerations, these comorbidities likely influenced the clinical outcomes in the authors' study.

How ought we to integrate these findings into routine clinical practice? The authors recommend a critical evaluation of the indications for primary prophylactic ICD implantation in elderly patients.^1^ Notably, individuals aged over 80 years reaped no discernible benefits from primary prophylactic ICD implantation, while a considerable number remained neutral.^1^ These neutral patients might experience appropriate ICD therapy during prolonged observation. Once more, assessing the equilibrium between the advantages and drawbacks of prophylactic ICD implantation proves pivotal in determining the appropriateness of ICD implantation within the elderly cohort.

## CONFLICT OF INTEREST STATEMENT

The authors declare no conflict of interest.
